# End-targeting proteomics of isolated chromatin segments of a mammalian ribosomal RNA gene promoter

**DOI:** 10.1038/ncomms7674

**Published:** 2015-03-27

**Authors:** Satoru Ide, Jerome Dejardin

**Affiliations:** 1 Inserm Avenir: ‘Biology of repetitive sequences’-Institute of Human Genetics, CNRS UPR1142, 141, rue de la Cardonille, Montpellier 34396, France

## Abstract

The unbiased identification of proteins associated with specific loci is crucial for understanding chromatin-based processes. The proteomics of isolated chromatin fragment (PICh) method has previously been developed to purify telomeres and identify associated proteins. This approach is based on the affinity capture of endogenous chromatin segments by hybridization with oligonucleotide containing locked nucleic acids. However, PICh is only efficient with highly abundant genomic targets, limiting its applicability. Here we develop an approach for identifying factors bound to the promoter region of the ribosomal RNA genes that we call end-targeting PICh (ePICh). Using ePICh, we could specifically enrich the RNA polymerase I pre-initiation complex, including the selectivity factor 1. The high purity of the ePICh material allowed the identification of ZFP106, a novel factor regulating transcription initiation by targeting RNA polymerase I to the promoter. Our results demonstrate that ePICh can uncover novel proteins controlling endogenous regulatory elements in mammals.

## Abstract (web summary)

The identification of factors involved in eukaryotic DNA regulation at specific genomic regions distinct technical challenges. Here, the authors describe ePICh, a method that allows for the efficient isolation of chromatin factors associated with complex low abundance targets within the large genome of mammalian cells.

## Graphical abstract

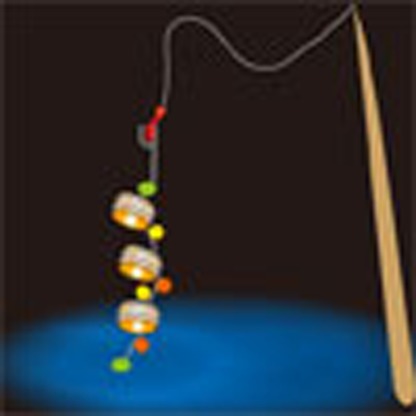

Eukaryotic DNA biology is supported by the biochemical activities of hundreds of protein factors, nucleic acids and small molecules that cooperate in a structure named chromatin. Defects in chromatin regulation have usually profound consequences on cellular identity or on the proliferative potential. Therefore, how these diverse types of players function at specific genomic regions and how their activities are controlled and integrated to drive biological reactions are critical questions that remain difficult to address experimentally. This difficulty is a consequence of limited information about the identity of all of the players acting at a specific genomic region at any given time.

To address this long-standing issue, attempts have been made for more than 30 years to purify specific chromatin loci^1^,^2^,^3^,^4^,^5^,^6^. However, most of these approaches usually fail in identifying new locus-specific proteins, indicating that locus-specific chromatin purification is a remarkably complicated biochemical challenge. First, the highly heterogeneous and dynamic nature of chromatin makes it difficult to maintain chromatin integrity *in vitro*, and second, the usually extremely low abundance of selected targets requires a very high degree of enrichment. Recent reports have described the identification of novel components after the affinity purification of specific chromatin proteins^7^. However, these approaches do not target loci directly but rather have relied on the use of antibodies to immunoprecipitate adaptor or endogenous proteins, and thus also purify protein complexes dissociated from the DNA or multiple loci bound by the target factor.

An alternative method is the proteomics of isolated chromatin segment (PICh) technique, which was developed to target chromatin containing simple DNA repeats in the mammalian genome^10^. In PICh, affinity purification relies on the specific hybridization of nucleic acid probes to selected targets and thus can be directly used to purify endogenous targets. PICh does not require the generation of a transgenic target or the expression of adaptor proteins or any prior knowledge about the identity of the bound proteins. To increase the stability of probe–chromatin interactions, PICh probes contain approximately 50% locked nucleic acid (LNA) residues. These LNA residues have an altered backbone that favours base stacking, thereby significantly increasing hybrid melting temperature^11^ and thus also increasing hybrid stability. To date, PICh has been shown to be effective with simple telomeric repeats and mouse pericentromeric repeats^12^, which are abundant targets in mammalian genomes. Adapting the PICh technique to isolate other chromatin regions harbouring more complex sequences, such as regulatory elements, would constitute a significant breakthrough in chromatin research.

Here, we identify the critical parameters for a successful PICh experiment and develop an improved version of the PICh method, in which chromatin segment ends are defined by a restriction enzyme and chromatin is solubilized using a *French* press. We called this method end-targeting PICh (ePICh). As the regulation of ribosomal RNA transcription is deeply linked to the proliferation of cancer cells, we used ePICh to purify an ~1-kb segment of chromatin from the ribosomal RNA (rRNA) gene promoter in order to identify factors that are important for proliferation. We identify more than 100 promoter-associated proteins, including most known rRNA promoter interactors. ePICh also identified zinc finger protein 106 (ZFP106) at the rRNA promoter and we show that this protein plays an important role in pre-rRNA synthesis by recruiting the RNA polymerase I machinery on the promoter region. Thus, ePICh allows the unbiased identification of new proteins bound *in vivo* to endogenous mammalian regulatory elements with complex sequences.

## Results

### Identification of key parameters for designing PICh probes

**Figure 1:**
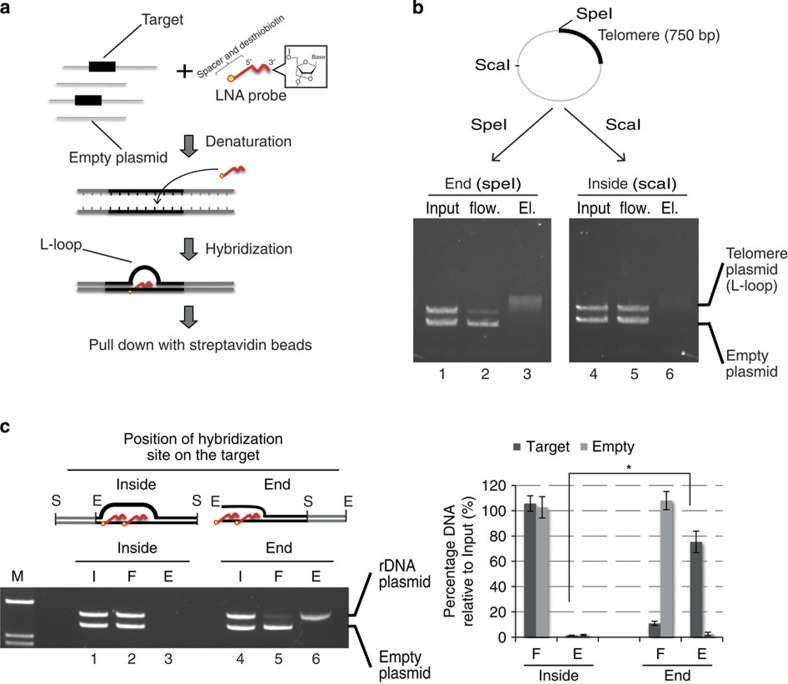
Rules for designing capture probes for the formation of stable oligo-DNA hybrids. (**a**) Outline of the plasmid pull-down assay. Linearized plasmids (grey line) with or without the target DNA sequence (black thick line) are mixed with LNA probes conjugated to spacers (straight red line) and desthiobiotin (yellow circle). After denaturation, the mixture is incubated at 37 °C for hybridization. Hybrids are captured using streptavidin beads. (**b**) Purification of the telomere sequence containing plasmid with the telomere-specific probe. Each fraction (Input, Flow-through (Flow.), Eluate (El.)) was analysed by agarose gel electrophoresis and EtBr staining. The positions of plasmid containing telomere DNA and the empty vector are indicated. Linear plasmid with telomere sequence after digestion with *Spe*I was used for an assay (lane 1–3 in the left panel), and linear plasmid digested by *Sca*I was used for the other assay (lane 4–6 in the right panel). The captured band is less intense and upshifted due to the presence of the 750-bp long L-loop. The plasmid map shows the location of telomere sequence and the two restriction sites used (*Spe*I and *Sca*I). (**c**) rDNA-containing plasmid and empty plasmid were cut in the backbone region with *Sac*I (S) to yield the target site for probes 1 and 2 inside the construct, or were digested with *Eco*RI (E) to yield the target site on the end of the fragment. (I, input; F, Flow-through; E, Eluate). Right panel: Histogram quantifying target capture (error bars represent standard deviations. Asterisks denote significance, *P<0.001 from a student-*t* test). Plasmid maps not on scale.

Locus-specific chromatin purification by PICh with a single probe containing LNA residues has been successfully used to isolate telomeric chromatin^10^. However, we failed to isolate chromatin from the rRNA gene (rDNA) promoter using the same protocol and several probes randomly targeting this sequence at multiple positions. To optimize probe design for efficiently capturing a complex target sequence, we established an *in vitro* assay to measure the capture of selected double-stranded DNA targets. Typically, our probes were 20–30 nucleotides (nt) long, comprised approximately equal amounts of LNA and DNA residues, and included a long spacer arm and a biotin analogue (desthiobiotin) at the 5′ end. The target DNA was first cloned into a plasmid vector, which was subsequently linearized by restriction digest. The linearized plasmid was then mixed with the capture probes and with an empty plasmid to monitor non-specific capture. After denaturation, the capture probes were allowed to bind to the target DNA, and the hybrid was isolated using streptavidin beads (Fig. 1a). In this assay, the telomere probe (24 nt) efficiently captured a plasmid containing a 750-bp telomere fragment linearized by digestion with *Spe*I, whereas the empty plasmid was not retrieved (Fig. 1b, left panel). Surprisingly, capture efficiency was dramatically decreased when the same target plasmid was digested with *Sca*I, positioning the telomere sequence in a more central location (Fig. 1b, right panel).

These results suggest that stable capture is achieved because a single region of several hundreds of nucleotides on one end of the DNA fragment is hybridized by multiple oligonucleotides, thus producing a large displacement loop (L-loop, because oligonucleotides contain LNAs) as evidenced by the band-shift observed in the eluates (Fig. 1b, lanes 3,6). Importantly, despite the presence of LNAs, the 750-bp long L-loop formed in a more central location is unstable, presumably because of the very high probability to re-anneal the duplex DNA on both sides of the displaced loop via branch migration^13^,^14^,^15^.

**Figure 2:**
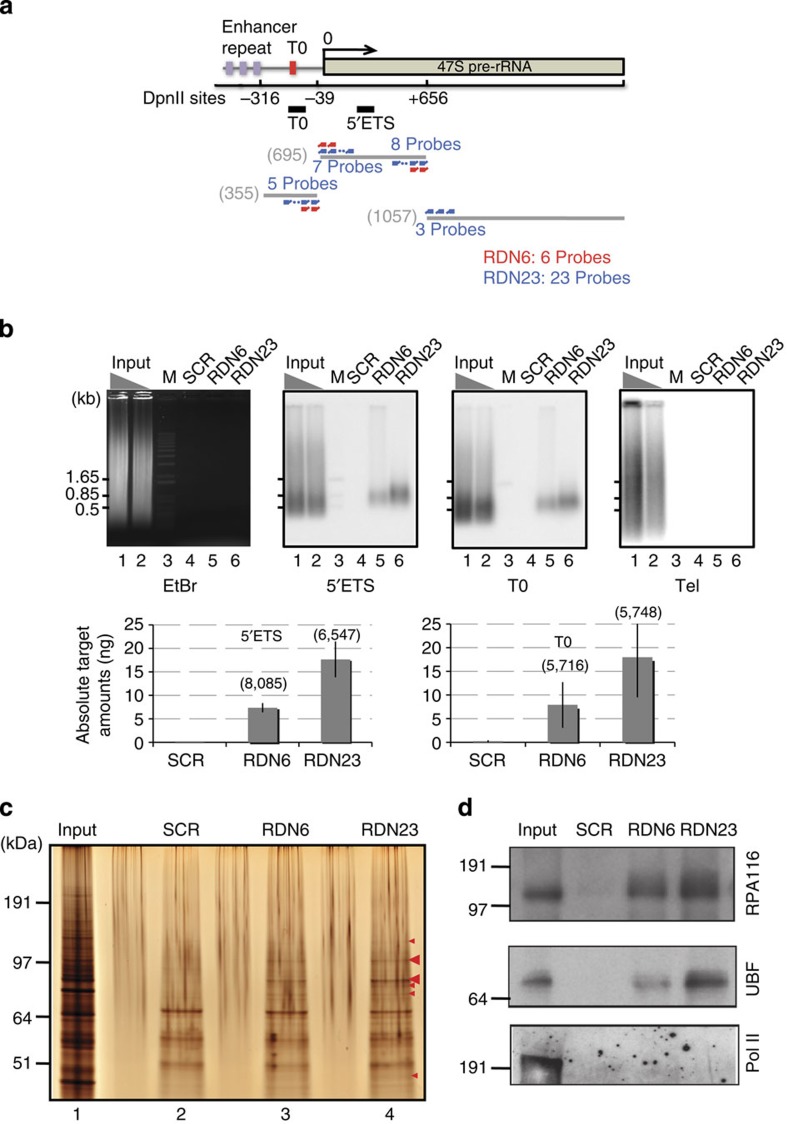
rDNA promoter chromatin purification. (**a**) The map of probe hybridization sites on the rDNA promoter region. An arrow: 47S pre-rRNA transcription initiation site. Scale bars (in bp) are shown below; 0 indicates the 5′ end of the pre-rRNA. Red bars: TTF-1-binding sites. Purple bars: repetitive enhancer elements are indicated. Black thick lines (T0 and 5′ETS) probes used in the Southern blots of **b** and Supplementary Fig. S2b. Grey lines: *Dpn*II fragments from the promoter region. The probes that are designed to bind around *Dpn*II sites are represented in red for ePICh with 6 probes (RDN6) and in blue for ePICh with 23 probes (RDN23). (**b**) DNA from ePICh material analysed by agarose gel electrophoresis. DNA was detected by EtBr staining (left panel) and quantified by Southern blotting with probes specific for the 5′ETS region, the TTF-1-binding site (see **a**) and telomere. Input represents 10 and 5% of the lanes 1 and 2 of the starting material. ePICh purified DNA using scrambled probe (lane 4), RDN6 probe (lane 5) and RDN23 probe (lane 6) were analysed. Bottom bar graph, absolute quantification of rDNA amount in ePICh material. The signal of the materials from ePICh with each probe were compared with a standard plasmid DNA containing the *Dpn*II fragment from 5′ETS and the *Dpn*II fragment from T0 shown in **a**. The numbers above the graph bars represent the relative enrichment of the analysed rDNA fragments compared with major satellites, as measured by real-time PCR. (**c**) Silver staining of proteins obtained from ePICh with scrambled, RDN6 and RDN23 probe sets in mouse erythrocytes leukaemia (MEL) cells. 10% of the ePICh material was analysed. Red arrowheads show specific bands detected using RDN6 and RDN23 probes. (**d**) Western blot analysis monitoring ePICh enrichment. The second biggest subunit of RNA polymerase I (RPA116) and UBF in materials from ePICh with each probe set were assayed. As a negative control for background enrichment, the RNA Polymerase II RPB1 subunit was probed. Input represents 0.0004% of the chromatin extracts. 5% of the ePICh material were loaded per lane.

To estimate the minimal length to obtain a stable end-positioned LNA-DNA hybrid, we next targeted a more complex sequence than telomeres: the murine rDNA promoter. We first designed two 24 nt consecutive probes targeting this region (to obtain a 48 bp compound hybrid) and performed the plasmid pull-down assay. When the 48-nt displaced loop was located inside the fragment (that is, forming branch migration points at both ends of the short L-loop), there was no detectable capture (Fig. 1c and Supplementary Fig. 1a). However, when the same sequence was positioned at the end of the DNA target (forming only one branch migration point on the 3′ end of the capture probes), the target fragment was efficiently captured (~80%, Fig. 1c and Supplementary Fig. 1a). We conclude that short hybrids (~50 bp) located at the end of a double-stranded DNA fragment remain stable during the purification process. In contrast, the same hybrids located inside the target are unstable, even when the invading molecules contain LNA residues. Moreover, the use of a single probe (24 nt hybrid) to target the end of the fragment did not allow the capture of duplex DNA (Supplementary Fig. 1b), suggesting that two consecutive probes (forming a 48 bp hybrid) are required for stable binding. This result also suggests that the nonspecific capture of DNA fragments through partial hybridization is highly unlikely to occur under PICh assay conditions. Finally, we found that the probe spacer position also significantly affects the capture efficiency, most probably because of steric hindrance. The yield was drastically reduced when the spacer moiety was close to the branch migration point (Supplementary Fig. 1c, lanes 4–6). Thus, as shown in Fig. 2a, probes should be designed to bind to the 3′ strand on both ends of the target.

From these experiments, we deduced a number of important rules for designing PICh probes: (i) the hybridization site should be located at the end of the target DNA fragment; (ii) at least two consecutive probes should be used for each targeted end and (iii) the probes should target the 3′ extremities at the end of both strands.

### Preparation of soluble chromatin for end-targeting by PICh

Sonication breaks formaldehyde crosslinked chromatin at essentially random positions and is a common and effective step to solubilize formaldehyde-crosslinked chromatin for downstream applications like chromatin immunoprecipitation (ChIP). The chromatin fragments used in PICh are also generated by sonication and thus do not have defined ends. This might explain why PICh can be used to efficiently capture telomeric chromatin fragments (in which the same motif repeat is found at any position, including at the ends) but not for retrieving targets with complex sequences. In fact, with the use of sonication to produce chromatin fragments with an average size of 2 kb, the probability of generating a fragment with an end that can hybridize to a specific probe is theoretically only 1/2,000 (5 × 10^−4^), which critically compromises the capture’s yield. To generate target fragments with defined ends, we treated formaldehyde-fixed nuclei with a restriction enzyme before chromatin solubilization. We used DpnII, a frequent 4 bp cutter that can digest DNA in formaldehyde-fixed chromatin samples^16^. The rDNA promoter region was only partially digested from crosslinked chromatin, with an efficiency of 45–70% depending on the restriction site (Supplementary Fig. 2a).

Next, we synthesized two consecutive probes targeting the 3′ sequence of the left end of the *Dpn*II fragment in the murine rDNA promoter region (at positions −39 to +654, Fig. 2a). To monitor capture efficiency, we tested the ability of these oligonucleotides to deplete the rDNA promoter segment from protein-free genomic DNA. For this, murine genomic DNA was digested with *Dpn*II, mixed with the probes, and used in the capture assay. As shown in Supplementary Fig. 2b, the rDNA promoter fragment was quantitatively depleted from the flow-through fraction and recovered in the eluate (Supplementary Fig. 2b, lanes 4,5). Thus, we were confident that these probes could efficiently hybridize to and capture the rDNA promoter from whole genomic DNA.

However, we were concerned that the long incubation of chromatin at 37 °C for digestion could partially reverse the protein–DNA crosslinks, leading to the capture of DNA, but not of associated proteins. Therefore, we analysed chromatin features after *Dpn*II digestion and solubilization using cesium chloride (CsCl) gradient ultracentrifugation analysis. This method separates fixed chromatin fragments by density, which allows monitoring the extent of protein–DNA interaction in fixed chromatin samples (Supplementary Fig. 3a). Naked DNA has the highest density and accumulates at the bottom of CsCl gradients, whereas free proteins have a much lower density and equilibrate to the top of the gradient. Chromatin, which is composed of DNA and proteins, equilibrates at intermediate densities and is therefore found in the middle of the gradient^17^. Surprisingly, chromatin solubilization by sonication (even without DpnII digestion) led to a major disruption of protein–DNA crosslinks at the rDNA promoter region. Indeed, a large proportion of the promoter signal was present in the densest fractions 1–3 (Supplementary Fig. 3b). This result was locus-specific because sonication did not quantitatively reverse protein–DNA crosslinks from bulk chromatin or from telomeres, as shown by the normal distribution of histone H3 and telomeric signal in these gradients.

We therefore used the *French* press (a high-pressure homogenizer) as an alternative method to solubilize *Dpn*II-digested chromatin. Using this method, most of the rDNA promoter signal was found in the same fractions as upstream binding factor (UBF), a protein that is known to bind to the rDNA gene *in vivo*, suggesting that protein–DNA crosslinks were maintained at the rDNA promoter (Supplementary Fig. 3c,d).

It has been reported that lower amounts of formaldehyde (0.25%) were necessary to solubilize nucleolar chromatin^18^. Thus, we also tested whether lower formaldehyde crosslinking would favour chromatin solubilization and ultimately, ePICh yield. As can be seen in Supplementary Fig. 4, most of the rDNA was protein free in this crosslinking condition, suggesting that only strong crosslinking allows to quantitatively maintain proteins on the DNA in the conditions we use for ePICh.

We conclude that *Dpn*II digestion does not detectably reverse protein–DNA crosslinks. Moreover, we have shown that the *French* press is a better tool than sonication for solubilizing formaldehyde-crosslinked chromatin while maintaining protein–DNA interactions at sensitive regions of the genome, such as the rDNA promoter.

In addition, the relative solubility of the rDNA promoter chromatin to the bulk chromatin seemed slightly higher when the *French* press was used (Supplementary Fig. 5).

### Purification of the rRNA gene promoter chromatin by ePICh

We next performed ePICh using three probe sets: negative control probes containing random (scrambled) sequences and sets of 6 (RDN6) or 23 (RDN23) probes that bind to the ends of several *Dpn*II fragments around the rDNA promoter region encompassing the 5′ external transcribed sequence (5′ ETS) and the TTF-1-binding site (Fig. 2a). We measured the ribosomal DNA yield and its relative enrichment over telomere DNA by Southern blotting analysis, which was used here as an abundant non-ribosomal DNA-negative control (Fig. 2b). ePICh using the scrambled probe did not provide any detectable signal; however, approximately 1% and 3% of rDNA from the input, corresponding to 2.5% and 6% of digested chromatin, were recovered using the RDN6 and RDN23 probe sets, respectively. On the other hand, the signals of telomeric DNA were below the detection limit (<10 pg; Fig. 2b), suggesting the high purity of ribosomal DNA in eluates with RDN6 and RDN23 probes. In addition, we evaluated the relative enrichment of the target to another abundant locus, the major satellite DNA (~4% of the mouse genome) by real-time PCR. Depending on the fragment analysed, the locus was enriched 5,700 to 8,000 fold over the major satellite (bracketed numbers in Fig. 2b), suggesting the very high purity of the ePICh material. The absolute amount of purified rDNA from 8 × 10^9^ cells was approximately 7 ng each of the 5′ ETS region and TTF-1-binding site (~15 fmol) using RDN6 probes or 17.7 ng 5′ ETS and 17.9 ng TTF-1-binding regions (~40 fmol) using RDN23 probes (Fig. 2b, lower panel). As proteins are quantitatively crosslinked to DNA in PICh conditions (Supplementary Fig. 3c), associated proteins should be present in nanogram quantities. The protein profile of these purified fractions was thus analysed by silver staining, and indeed several specific bands were detected for each rDNA probe mixture (Fig. 2c, red arrowheads). As a positive control to test for the specific enrichment of rDNA promoter-binding proteins, we monitored the presence of UBF and the second biggest subunit of RNA polymerase I (RPA116) in ePICh preparations by immunoblotting (Fig. 2d). Both proteins were highly enriched. As a negative control, we verified that RNA polymerase II was not enriched in these samples (Fig. 2d). Importantly, when the original PICh protocol was employed with the same 23 probes set, none of these proteins were detected in the eluates (Supplementary Fig. 6a), despite a significant capture of ribosomal DNA (Supplementary Fig. 6b). This is consistent with our data showing that sonication disrupts protein–DNA crosslinking at the rDNA promoter (Supplementary Fig. 3b,d). Moreover, we also failed to detect enrichment in nucleolar proteins when we used the *Dpn*II-digested, sonicated chromatin (data not shown), indicating that sonication should be avoided when analysing the protein content of rDNA chromatin. This also suggests that ePICh is a better method for affinity purifying low-abundance chromatin with a complex DNA sequence.

### The chromatin proteome of the rRNA gene promoter

**Figure 3:**
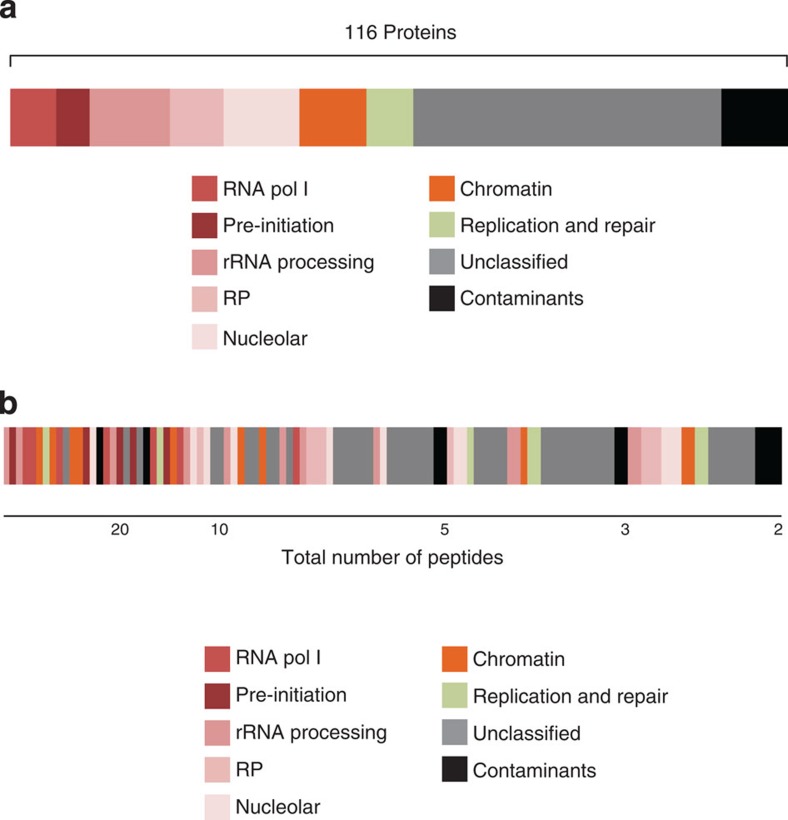
rDNA gene promoter chromatin proteome. (**a**) Categorization of the 116 proteins enriched in ePICh with both RDN6 and RDN23 into nine functional groups: specific subunits of the RNA polymerase I core complex (RNA pol I), Pre-initiation complex for pol I transcription (pre-initiation), ribosomal RNA processing and modification proteins (rRNA processing), ribosomal proteins (RP), proteins localized in the nucleolus (Nucleolar protein), chromatin regulatory protein (Chromatin), proteins involved in DNA replication and repair (Replication and Repair), unclassified proteins (Unclassified), streptavidin and mitochondria proteins (Contaminants). (**b**) List of proteins enriched at the rDNA promoter according to the number of identified peptides and arranged in descending order. Scale bars (in peptide number) are shown below.

Next, proteins purified by ePICh were analysed by mass spectrometry (MS). We identified 588 and 592 proteins that were pulled down by the RDN6 and RDN23 probe mixes, respectively. Proteins identified on the basis of a single peptide were discarded from the analysis, leaving 116 proteins reproducibly enriched in both the RDN6 and RDN23 preparations (at least three times more peptides than with the scrambled probe; Fig. 3a and Supplementary Data 1). These 116 proteins were categorized into nine groups: RNA Pol I subunits, pre-initiation complex for RNA Pol I, rRNA processing proteins, ribosomal proteins, nucleolar proteins, chromatin regulatory factors, DNA replication and repair proteins, unclassified proteins and typical PICh contaminants, such as mitochondrial proteins (carboxylases and partners), which are naturally biotinylated. Sorting the enriched proteins according to the normalized spectral abundance factor in the MS analysis revealed that nine of the ten top proteins are known to be associated with rDNA and involved in rDNA gene transcription (Fig. 3b and Table 1). This shows that ePICh can efficiently and specifically purify rDNA promoter chromatin.

rDNA-interacting proteins include most subunits of RNA Pol I (six out of eight sub-units) as well as UBF, treacle protein (TCOF1), FACT complex and topoisomerase I (Table 1)^19^,^20^,^21^,^22^. In addition to major RNA Pol I-specific subunits (Supplementary Table 1), ePICh identified proteins specifically associated with the promoter site and not with other rDNA regions, such as members of the RNA Pol I pre-initiation complex, the SL1 complex and TTF-1 (Supplementary Table 2). The highly specific binding of these proteins is required for precise regulation of transcription initiation within the targeted region^23^,^24^. Moreover, these proteins are singly bound to this target, suggesting that ePICh preparations are sufficiently pure to enable proteins singly bound to a complex 1 kb promoter region to be readily identified, without the need for more sophisticated MS approaches or for the computational filtering of contaminants. However, as with any purification procedure, false positive associations cannot be excluded, and any newly identified factor should be independently validated. We conclude that ePICh is an appropriate method for the proteomic characterization of a 1-kb chromatin fragment containing a complex DNA sequence present at approximately 200 copies in mammalian cells.

### ePICh analysis of active and silent promoters

In the mouse genome, the rDNA loci are composed of ~200 copies of the rDNA gene. A fraction is actively transcribed while other copies are silent. ePICh cannot discriminate between the two states. Active rDNA promoters form open chromatin and are hypomethylated at CpG sites, while silent units are methylated at CpG sites^25^. Our MS analysis identified multiple proteins known to be associated with active rDNA promoters (Fig. 3a and Supplementary Data 1). However, ePICh did not retrieve the TTF-1-interacting protein 5 (TIP5), which is typically associated with inactive rDNA copies^26^,^27^. This suggested that ePICh may have preferentially captured active rDNA promoters.

To examine this possibility, we compared the CpG methylation content of captured DNA and input chromatin DNA using the methylation-sensitive restriction enzyme *Hpa*II. In the purified DNA, *Hpa*II-resistant DNA reflects the CpG-methylated fraction (and thus inactive copies of the rDNA promoter), which is amplifiable by PCR. This method was originally developed on the rDNA analysed by ChIP to characterize the chromatin features of the plant rDNA locus and was named ‘ChIP-Chop’^28^. Our ‘PICh-Chop’ experiment showed that methylated DNA accounts for approximately 30% of ePICh-purified material. This is comparable to the percentage of methylated rDNA in the input (~ 40%, Supplementary Fig. 7). These results suggest that ePICh purifies active and inactive rDNA promoters with comparable efficiencies. The absence of TIP5 in ePICh preparations may therefore indicate that interaction of TIP5 with the promoter region is too transient to be captured by this method.

### ZFP106, a functionally important rRNA-controlling protein

**Figure 4:**
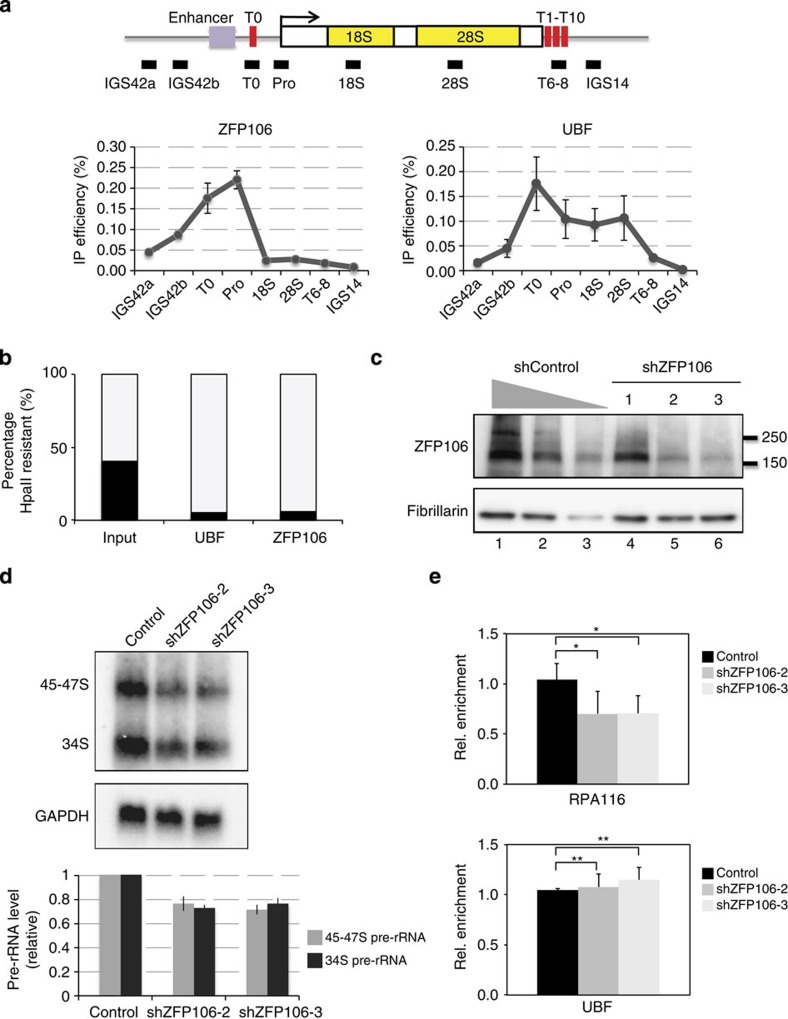
Validation of ZFP106 as a true and important rDNA promoter-binding protein. (**a**) Chromatin immunoprecipitation of ZFP106 using anti-ZFP106 antibody and anti-UBF antibody. Immunoprecipitated (IP) DNA relative to input were quantified by real-time quantitative PCR. Top: Map of regulatory sequence elements on rDNA unit. The thick black line shows the position of PCR amplicons. (**b**) ChIP-chop experiment to measure DNA methylation level 275 bp downstream of the rDNA promoter in IP DNA with anti-UBF, anti-ZFP106 and input DNA. See the details in Supplementary Fig. 5. (**c**) Western blotting analysis for ZFP106 and fibrillarin after knockdown by three independent shRNA constructs. Whole protein extract after knockdown with shControl (lane 1: 100%, lane 2: 50%, lane 3: 20% of the extract) and the protein extract in the cells expressing three shRNA for ZFP106 were analysed (lanes 4–6). (**d**) Northern blotting analysis of pre-rRNA levels. Pre-RNA, 45–47S rRNA and 34S rRNA were detected by the ETS3 oligoprobe and glyceraldehyde 3-phosphate dehydrogenase (GAPDH) messenger RNA (mRNA) was detected by a specific oligoprobe. The bars represent pre-rRNA levels (45–47S and 34S) normalized to GAPDH mRNA from three experiments. (**e**) ChIP analysis of the second biggest subunit of RNA polymerase I (RPA116) and UBF on rDNA in the cells expressing shControl and two shRNA for *ZFP106*. Immuno precipitated DNA relative to input were quantified by real-time quantitative PCR for the rDNA promoter region (Pro) and then normalized to control reactions from shControl-transfected cells. Three independent experiments were performed and error bars represent standard deviation. Stars denote significances, *P<0.01, and **P>0.2 from a Student’s *t*-test.

Identifying factors specifically involved in cellular proliferation is critical for developing new therapeutic strategies for proliferative diseases. Because ribosome production is necessary for cell proliferation, newly identified factors that control rRNA transcription may represent novel targets for anticancer therapy. Besides RNA polymerase I subunits, only a handful of factors are known to be specifically involved in rRNA transcription (UBF, SL1 and TTF1). In addition to these components, many proteins (52) that we found to be enriched at the rDNA promoter are not known to be involved in the transcriptional control of rRNA genes. Here, we focused on one of these enriched proteins, the zinc finger protein 106 (ZFP106), a nucleolar protein reported to interact with testis-specific gene 118 (TSG118)^29^, another protein identified in ePICh preparations. We investigated ZFP106 binding to the rRNA promoter region by ChIP using an anti-ZFP106 antibody, and used UBF ChIP as a positive control for enrichment. ZFP106 exclusively localized to the rDNA promoter region, thus validating ePICh. In contrast, UBF had a much broader distribution in this region, consistent with previous reports^18^,^30^ (Fig. 4a).

To examine whether ZFP106 selectively binds to active or silent copies of rDNA, we performed ChIP-Chop experiments using ZFP106 and UBF antibodies. As shown in Fig. 4b, the rDNA enriched by ZFP106 ChIP was sensitive to *Hpa*II digestion (94% digested), similar to the rDNA bound by UBF (95% digested). This shows that ZFP106 occupies hypomethylated rDNA promoters, thus suggesting that ZFP106 binds to the active rDNA promoter.

To explore the role of ZFP106 in rRNA synthesis, we used lentivirus-expressed short hairpin (sh)RNA to deplete this protein from mouse erythrocyte leukaemia (MEL) cells, and then measured the amount of pre-rRNA transcript by northern blotting. Efficient ZFP106 knockdown by the transduction of two independent shRNA constructs was verified by immunoblotting (Fig. 4c, lanes 5,6). This was associated with a 30% reduction in the levels of 45–47S and 34S pre-rRNA (Fig. 4d). Consistently, RNA polymerase I association to the rDNA promoter was decreased to similar extents: there was a 30% reduction of RPA116 association by ChIP in the two independent shZFP106 cell lines. Interestingly, ZFP106 knockdown did not measurably affect UBF association (Fig. 4e). This result indicates that ZFP106 binding to the rDNA promoter facilitates the production of pre-rRNA transcripts by recruiting RNA polymerase I to the rRNA gene transcription start site. Thus, ePICh is useful in identifying novel factors controlling rRNA expression.

## Discussion

We have demonstrated that nucleic acid hybridization-based approaches for locus-specific chromatin purification allow the unbiased identification of proteins associated with an endogenous regulatory element of the mammalian genome. The plasmid pull-down assay (Fig. 1) suggests that chromatin capture by PICh occurs via hybridization to the ends of the target fragment and requires the binding of at least two consecutive probes containing LNA residues. When telomeres or other simple repeats are targeted, all of the relevant fragments obtained by sonication harbour hybridization sites at their ends and are thus stably captured by a probe matching those repeats. When more complex sequences (such as the rDNA promoter) are targeted, the ends of the target should be defined and probes designed to specifically hybridize to those ends. Another critical point to consider in any locus-specific chromatin purification strategy is the signal-to-noise (S/N) ratio. For ePICh of the rDNA promoter, the S/N ratio in the starting material corresponded to ~200 copies of a 1-kb promoter region compared with the whole mouse genome (S/N=2.0 × 10^5^ bp/2.5 × 10^9^ bp=1/10,000). Thus, rDNA-bound proteins must have been enriched 10,000 times to be found in higher amounts than background. In fact in ePICh samples, we measured relative rDNA enrichments of up to 8,000 when compared with major satellites (Fig. 2b). These S/N ratios almost correspond to 1 kb of a single-copy locus from the yeast genome (10^3^ bp/1.2 × 10^7^ bp). For comparison, an excellent ChIP enrichment value is usually two orders of magnitude lower (~100), suggesting the use of antibodies to precipitate crosslinked chromatin may not be appropriate for proteomics studies of low abundance targets. Following this logic, the purification of ~1 kb single-copy regions from the mammalian genome would require an enrichment factor of 10^6^-fold. We currently do not know whether PICh or ePICh can achieve such enrichment values. With an enrichment factor of 10,000, only 1% of the purified mixture would correspond to proteins bound to a single copy locus (in other words, 99% would be contributed by contaminants). Nonetheless, in organisms with smaller genomes, both endogenous repeated loci and endogenous single loci are suitable targets for PICh-based chromatin purification approaches.

Using ePICh, we identified 116 proteins enriched at the promoter region of the murine rDNA gene. The majority (80/116) are present in the very large Nucleolar Proteome Database (NOPdb ver.3), a database that contains ~4,500 proteins, thus validating ePICh. Conversely, we did not identify TIP5 (also absent in NOPdb) suggesting that ePICh may not be sensitive enough to capture transient associations. Interestingly, some proteins not listed in the NOPdb^31^,^32^ were detected by ePICh, for example, the components of the SL1 complex, which is critical for RNA Pol I transcription. This suggests that promoter-bound proteins, which are undetectable by MS in whole nucleolar preparations, are enriched by ePICh. Thus, ePICh is a suitable method for finely dissecting the local chromatin content on rDNA. The use of ePICh in nutrient-depleted conditions, or to screen anticancer drugs will provide critical insights into how the rRNA gene is controlled. The discovery that ZFP106 is directly involved in this pathway illustrates the potential of ePICh. We believe this approach will open new avenues in cancer therapy and hold promises to characterize the chromatin proteome from other regulatory elements like endogenous retroviral sequences, or elements specifically involved in human diseases like the D4Z4 repeat, whose misregulation via unclear mechanisms causes Fascio Scapulo Humeral Dystrophy^33^.

## Methods

### ePICh

MEL cells were grown in Dulbecco’s modified Eagle’s medium supplemented with 10% fetal bovine serum and 2 mM L-glutamine. For each purification, 2 l of MEL cells (at 4 × 10^6^ cells per ml, i.e., 8 × 10^9^ cell equivalents) grown in a roller bottle were crosslinked with 3.7% formaldehyde in phospate-buffered saline (PBS) for 30 min at room temperature (RT). After washing four times with PBS, cells were transfered in sucrose buffer (0.3 M sucrose, 10 mM HEPES-NaOH, pH 7.9, 1% Triton X-100, 2 mM MgOAc) and then lysed with 20 strokes of a Dounce homogenizer. Chromatin was pelleted by centrifugation at 3,200*g* for 10 min at 4 °C. The pellet was resuspended in the same volume of glycerol buffer (25% glycerol, 10 mM HEPES-NaOH, pH 7.9, 0.1 mM EDTA, 0.1 mM EGTA, 5 mM MgOAc), and then frozen in liquid nitrogen and stored at −80 °C or used immediately for ePICh., ePICh was carried out according to a modified PICh protocol. The chromatin pellet was resuspended in an equal volume of PBS containing 0.5% Triton X-100 and 1 mg ml^−1^ RNase (Qiagen) and mixed at 4 °C overnight. After washing with restriction digest buffer (50 mM Bis-Tris-HCl, pH6.0, 100 mM NaCl, 10 mM MgCl_2_, 1 mM dithiothreitol), the pellet was thoroughly resuspended in the same buffer containing *Dpn*II (10,000 units) and incubated at 37 °C for 4 h with shaking at 1,200 r.p.m. EDTA was added to a final concentration of 11 mM, and the sample was rocked at RT for 5 min. The pellet was washed three times with PBS and twice with LB3JD buffer (10 mM HEPES, pH 7.70, 100 mM NaCl, 2 mM EDTA, 1 mM EGTA, 0.2% SDS, 0.1% Sarkosyl, 1 mM phenylmethylsulphonyl fluoride). After centrifugation at 3,000*g* for 8 min, the pellet was resuspended in a 1.5 × volume LB3JD buffer and then passed three times through a French press at 25,000 p.s.i. at RT. The chromatin solution was centrifuged at 3,000*g* for 6 min, and the supernatant was re-centrifuged at 20,000*g* for 15 min at 4 °C to remove all debris. The chromatin sample was heated at 58 °C for 5 min and then incubated overnight at 4 °C with a one-tenth volume of streptavidin agarose beads pre-equilibrated with LB3JD. The mixture was applied to Sephacryl S-400-HR spin columns. After centrifugation at 20,000*g* for 15 min, SDS was added to the supernatant to a final concentration of 0.2%, along with the LNA probes. Approximately 37.5 mg chromatin (in a 15 ml volume) was hybridized to each probe (scrambled, RDN6 and RDN23). The total concentration of each probe set was <0.1 μM. Hybridization was performed by incubation at 25 °C for 3 min, 74 °C for 7 min, 37 °C for 1.5 h, 70 °C for 5 min and 37 °C for 1.5 h. The sample was then centrifuged at 20,000*g* for 15 min, and the supernatant was diluted with 1 volume of MilliQ water and added to MyONE C1 magnetic beads (Invitrogen) pre-equilibrated with LBJD (1 ml for 1,200 pmol probes). The sample was incubated at RT for 2 h. Beads were then equally divided between two tubes and washed seven times with 10 ml of LB3JD and twice with 1 ml of LB3JD in a Protein Lobind tube (Eppendorf) at RT. As an additional washing step, beads were incubated with shaking in LB3JD containing 30 mM NaCl at 42 °C for 10 min, in LB3JD containing 10 mM NaCl at 42 °C for 10 min, and then in LB3JD containing 10 mM NaCl at 52 °C for 10 min. Finally, the beads were resuspended in 1 ml elution buffer and incubated with shaking at 37 °C for 1 h. For protein analysis of the eluate, trichloroacetic acid (18% final) precipitation was performed, and the pellet was incubated with 80 μl crosslinking reversal solution (250 mM Tris-HCl (pH 8.8), 2% SDS, 1 M 2-mercaptoethanol) at 99 °C for 30 min. Proteins from 65% of the eluate from ePICh with scrambled, RDN6 and RDN23 probes were separated using a 12% Bis-Tris acrylamide pre-cast gel (Invitrogen), and the gels were stained with colloidal blue for MS analysis or silver stained (Silver Quest kit, Invitrogen) for detecting unique bands purified by ePICh using rDNA probes, or subjected to western blot analyses with an anti-RPA116 (kind gift from Ingrid Grummt), anti-UBF (sc-13125, Santa Cruz Biotechnology) or anti RPB1 (8WG16, Covance MMS126R) antibodies. For protein identifications, gel lanes were cut into regions according to the banding pattern and subjected to MS analysis (Taplin Mass Spectrometry Facility). For analysis of DNA purified by ePICh, the eluate was incubated at 65 °C overnight in 50 mM Tris-HCl, pH 7.5, containing 200 μg ml^−1^ proteinase K and 2% SDS. After phenol–chloroform extraction and ethanol precipitation, the pellet was resuspended in TE buffer containing 10 μg ml^−1^ RNase. Agarose gel electrophoresis and Southern blotting were performed using the 5′ ETS and T0 probes.

### Plasmid pull-down assay

For each assay, 200 ng linearized plasmids (the plasmid containing the target DNA and the empty vector) were resuspended in LB3JD buffer containing 0.5 μM LNA probes. The mixture was denatured at 90 °C for 2 min and then incubated at 37 °C for 30 min. Twenty-five microlitres of MyONE C1 beads was added and incubated at RT for 30 min with shaking. Beads were then immobilized on a magnetic stand, and the supernatant was collected as the flow-through. Beads were washed five times with 1 ml of LB3JD at RT, incubated in LB3JD at 37 °C for 5 min, resuspended in LB3JD containing 12.5 mM D-biotin (Invitrogen), and incubated at 65 °C for 15 min for elution. One-tenth volumes of the input, flow-through and elution fraction were analysed by agarose gel electrophoresis, followed by ethidium bromide staining. Band intensities in the flow-through and elution fractions were quantified relative to the input in three independent experiments.

### Plasmid construction

The murine rDNA promoter region was PCR-amplified using specific primers (mpro-p1, 5′- GGAATTCGATCTTTTCTATCTGTTCCTATTGG -3′; mpro-p2, 5′- CGCGAGCTCTAAGAGTGAGCAACGACGCGCA -3′) from genomic DNA and cloned into the *Sac*I and *Eco*RI sites of the pGEM-T easy vector. The plasmid containing the region between −39 and +656 of the rRNA gene promoter was named mpro1 and used for the plasmid pull-down assay (Fig. 1c and Supplementary Fig. 1b). A derivative of the mpro1 plasmid, mpro2, in which the SpeI recognition sequence (ACTAGT) was inserted into the site at 9 bp downstream of the transcription start site, was constructed to examine the effect of probe spacer position on capture efficiency (Supplementary Fig. 1c).

### Equilibrium sedimentation of chromatin by CsCl gradient ultracentrifugation

After solubilization, the clarified lysate was mixed with 5 ml of LB3JD containing 2.84 g of CsCl and spun in 5 ml Beckman Ultra-Clear centrifuge tubes for 68 h at 20 °C (195,000*g*). Twelve 400 μl fractions were collected after centrifugation and numbered from N1 (bottom of the tube) to N12 (top of the tube). Fractions were dialysed against 10 mM Tris-HCl pH8.0, containing 1 mM EDTA and 0.5 mM EGTA. For DNA analysis, the cross-links were reversed by overnight incubation with 0.2 mg ml^−1^ proteinase K at 65 °C, followed by phenol–chloroform extraction and ethanol precipitation. DNA samples were analysed by Southern blotting using radiolabelled 5′ ETS or oligo DNA [(TTAGGG)_9_] probes. For protein analysis, fractions were precipitated using trichloroacetic acid and treated with crosslinking reversal solution at 99 °C for 30 min (as described in for ePICh). Proteins were analysed by western blotting using anti-UBF (1/1,000) and histone H3 (ab1791, Abcam, 1/5,000) antibodies.

### Chromatin immunoprecipitation

ChIP was performed using 2 μg of anti-UBF (sc-9131, Santa Cruz Biotechnology) and 2 μg of anti-ZFP106 (A301–527 A, Bethyl Laboratories) and 2 μl of anti-RPA116 (gift from Ingrid Grummt) antibodies and corresponding amount of anti-IgG rabbit antibody (Millipore) as a control, according to the method of Nemeth *et al*.^30^ Quantitative real-time PCR was performed using a LightCycler (Roche) and the SYBR Green detection system. Standard curves for relative quantifications with each primer sets (sequences described in Supplementary Data 2) were obtained from fivefold dilutions of the input sample. The amount of DNA in the precipitates of IgG versus specific antibodies differed by at least two orders of magnitude. rDNA ratios in the immunoprecipitates versus input chromatin were calculated as IP efficiencies in Fig. 4a. rDNA ratios in the immunoprecipitates versus input chromatin in the cells expressing shRNA for ZFP106 were normalized to the values obtained from the shControl-transfected cells in Fig. 4e.

### PICh-chop and ChIP-chop experiments

DNA purified by ePICh and by ChIP using 2 μg of anti-ZFP106 and anti-UBF antibodies was subjected to restriction digest with *Hpa*II or *Msp*I at 37 °C overnight before quantitative real-time PCR analysis. Digested DNA and an equal amount of undigested purified DNA was amplified by quantitative PCR using the primers shown in Supplementary Data 2. The amount of *Hpa*II-resistant, *Msp*I-sensitive PCR product indicates the level of rDNA promoter methylation, whereas the difference between the undigested and HpaII-digested signal indicates the proportion of unmethylated rDNA promoters. The presence of a MspI-resistant signal indicates that some of the purified DNA contains nicks or a single-stranded DNA region at the restriction site. Results are depicted as the percentages of methylated DNA, unmethylated DNA, nicked DNA and single-stranded DNA purified by RDN6 probes in ePICh or by antibodies in the ChIP experiment.

### Construction of shRNA plasmids and infection

pLKO-hygro-shZfp106 vectors were constructed as recommend by Addgene, and shRNA sequences were selected using siDIRECT web-based software^34^. Lentivirus particles were produced in 293T cells by the calcium phosphate method, according to the protocol recommended by Addgene. MEL cells were infected by the lentiviruses and hygromycin selection was initiated 1 day after infection using a final concentration of 500 μg ml^−1^. Infected cells were passaged every 2 days after selection, and stored as stocks 7 days after infection.

### Northern blotting to detect pre-rRNA

Total RNA was isolated using TRIzol (Invitrogen) from MEL cells after Zfp106 knockdown. Formaldehyde–agarose gel electrophoresis and northern blotting using oligonucleotide probes hybridizing to the 5′ ETS region of pre-rRNA or *GAPDH* messenger RNA (see Supplementary Data 2) were performed as described^35^, except that formamide-containing hybridization buffer (50% formamide, 0.25 M sodium phosphate (pH7.2), 7% SDS, 0.25 M NaCl) was used at 42 °C overnight instead of Denhardt’s solution at 60 °C. Ten micrograms of RNA were separated in 1.0% formaldehyde-agarose gel electrophoresis and blotted onto Hybond-N+ nylon membrane (GE healthcare) by neutral transfer system. Oligonucleotide probes were end-labelled with T4 polynucleotide kinase (New England Biolabs) and (γ-32P) ATP (Perkin Elmer) and purified by MicroSpin G-25 column (GE healthcare). Prehybridization and hybridization were performed in formamide-hybridization buffer. The filters were washed in 2XSSC/0.1% SDS for 5 min at room temperature twice and for 10 min at 70 °C. Intensities of the pre-rRNA and *GAPDH* bands were analysed with Typhoon FLA 9500.

## Author contributions

S.I. and J.D. conceived the project and designed the experiments. S.I. performed all experiments except MS experiments (Taplin MS facility) and qPCR quantifying enrichment fold of rDNA region in elution of ePICh (J.D.). S.I and J.D. wrote the manuscript.

## Additional information

**How to cite this article:** Ide, S. & Dejardin, J. End-targeting proteomics of isolated chromatin segments of a mammalian ribosomal RNA gene promoter. *Nat. Commun.* 6:6674 doi: 10.1038/ncomms7674 (2015).

## Supplementary Material

Supplementary Figures and Supplementary Tables Supplementary Figures 1-7 and Supplementary Tables 1-2

Supplementary Data 1 Raw MS data from PICh experiments

Supplementary Data 2 Oligonucleotides (ePICh probes, PCR and shRNA oligonucleotides used in this study).

## Figures and Tables

**Table 1:** The top ten proteins sorted by NSAF in the mixture obtained from ePICh with RDN23

**Name**	**Annotation**	**Total number of peptides**			**NSAF**	
		**RDN6**	**RDN23**	**SCR**	**RDN6**	**RDN23**
TCOF1	Treacle protein	280	308	81	0.090	0.087
UBTF	Upstream-binding factor 1	163	174	13	0.090	0.083
NOLC1	Nucleolar and coiled-body phosphoprotein 1	66	80	12	0.040	0.043
CD3EAP	DNA directed RNA polymerase I subunit RPA34	17	32	0	0.018	0.030
TOP1	DNA topoisomerase I	40	47	8	0.022	0.023
CFL1	Cofilin 1	12	10	3	0.031	0.023
LYAR	Cell growth regulating nucleolar protein	17	22	2	0.019	0.021
POLR2E	DNA-directed RNA polymerase I, II and III subunit	7	11	0	0.014	0.020
SSRP1	FACT complex subunit SSRP1	39	36	4	0.023	0.019
SUPT16H	FACT complex subunit SPT16	45	48	7	0.018	0.017

Normalized spectral abundance factor (NSAF) is shown to account for the fact that larger proteins statistically contribute more peptides/spectra. NSAF is calculated as the ratio of the number of spectral counts to the protein’s length and then normalized against the sum of all the ratio of spectral counts to the protein length. Total number of peptides identified in ePICh experiments with 6 probes (RDN6), 23 probes (RDN23) or the scrambled probe (SCR) are shown. Obvious contaminants from the beads (streptavidin proteins), which have a high NSAF were removed from this list.
