# Supplementary material for: End-targeting proteomics of isolated chromatin segments of a mammalian ribosomal RNA gene promoter
Source: Nat Commun. 2015 Mar 27;6:6674. doi: 10.1038/ncomms7674 (PMC4389260; doi:10.1038/ncomms7674)
Supplement: Supplementary Figures and Supplementary Tables — Supplementary Figures 1-7 and Supplementary Tables 1-2 [file ncomms7674-s1.pdf]

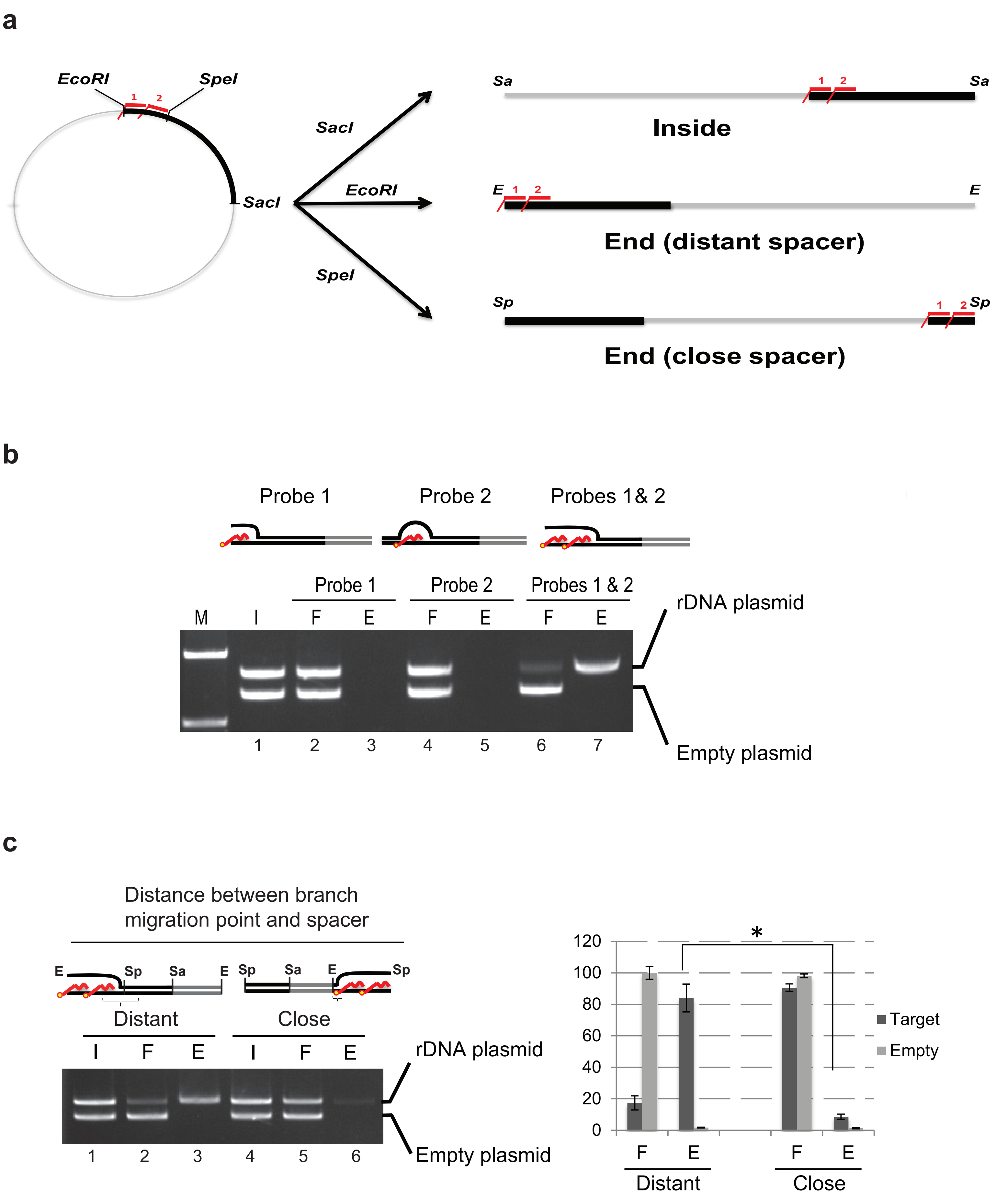
a
EcoRI
SpeI
1
2
SacI
SacI
EcoRI
SpeI
Sa
Sa
Inside
E
E
End (distant spacer)
Sp
Sp
End (close spacer)
b
Probe 1
Probe 2
Probes 1& 2
Probe 1
Probe 2
Probes 1 & 2
M
I
F
E
F
E
F
E
rDNA plasmid
Empty plasmid
1
2
3
4
5
6
7
c
Distance between branch migration point and spacer
E
Sp
Sa
E
Sp
Sa
E
Sp
Distant
Close
I
F
E
I
F
E
rDNA plasmid
Empty plasmid
1
2
3
4
5
6
120
100
80
60
40
20
0
*
Target
Empty
F
E
F
E
Distant
Close

Figure S1

Supplementary Figure 1 | Plasmid pulldown assays. (a) Restriction digestion strategy to yield the same target hybrid located at three different position in the duplex DNA of plasmid containing the region between −39 and +656 of the rRNA gene promoter. **(b)** The hybrid must be at least ~50 bp long for stable capture. A 48nt target sequence was partially targeted with one probe (Probe 1) or the other (Probe 2) or fully targeted using a combination of both (Probes 1 & 2). (I, input; F, flowthrough; E: eluate). **(c)** Distance between the spacers on LNA probes and the branch migration fork on the DNA duplex region influences capture (see the diagram). rDNA-containing plasmid DNA and empty plasmids were cut by EcoRI or by SpeI to generate targets which have a terminal localization of the same hybridization site either with the branch migration point close to the 3' end of the capture probes (EcoRI) or close to the 5' spacer position (SpeI). Right panel: Histogram quantifying target capture (error bars represent standard deviations. Stars denote significances, * $p<0.001$ from a student-t test).

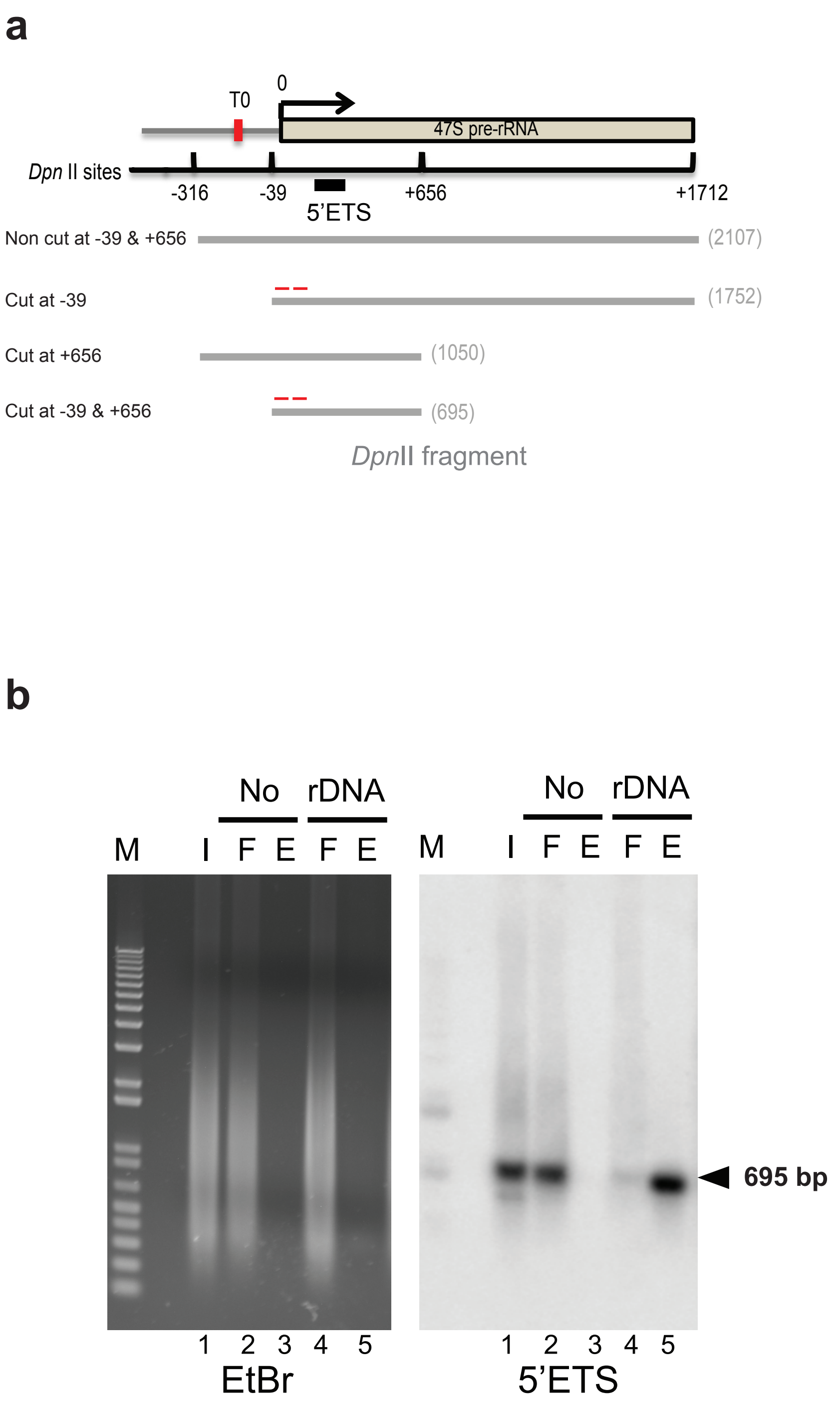
a
T0
0
47S pre-rRNA
Dpn II sites
-316
-39
+656
+1712
5'ETS
Non cut at -39 & +656
(2107)
Cut at -39
(1752)
Cut at +656
(1050)
Cut at -39 & +656
(695)
DpnII fragment
DpnII partial digestion pattern
6000 (bp)
2000~
1752
1000~
695
rDNA
No
DpnII
1.65
0.85
1
2
b
No
rDNA
M
I
F
E
F
E
1
2
3
4
5
EtBr
No
rDNA
M
I
F
E
F
E
695 bp
1
2
3
4
5
5'ETS

Figure S2

Supplementary Figure 2 | Restriction digestion of the crosslinked rDNA chromatin and the capture of rDNA region from genomic DNA by LNA probes.

(a) Partial DpnII digestion of formaldehyde crosslinked chromatin Left panel: DpnII restriction map around the rRNA promoter region.. Right panel: pattern of digested DNA before (lane1) and after DpnII action (lane2) (Southern hybridization with the 5' ETS probe). **(b)** Genome pull-down assay with RDN probes. Murine genomic DNA was digested with DpnII and mixed without or with the two LNA probes (RDN-m554, RDN-m564) bound to the end of DpnII fragment (-39). I, input (lane 1), F, flow through (lane 2 & 4), E, elution (lane 3 & 5) were analyzed by agarose gel electrophoresis and southern blotting with radiolabeled 5' ETS probe. Arrowhead shows the position of target DNA (695bp).

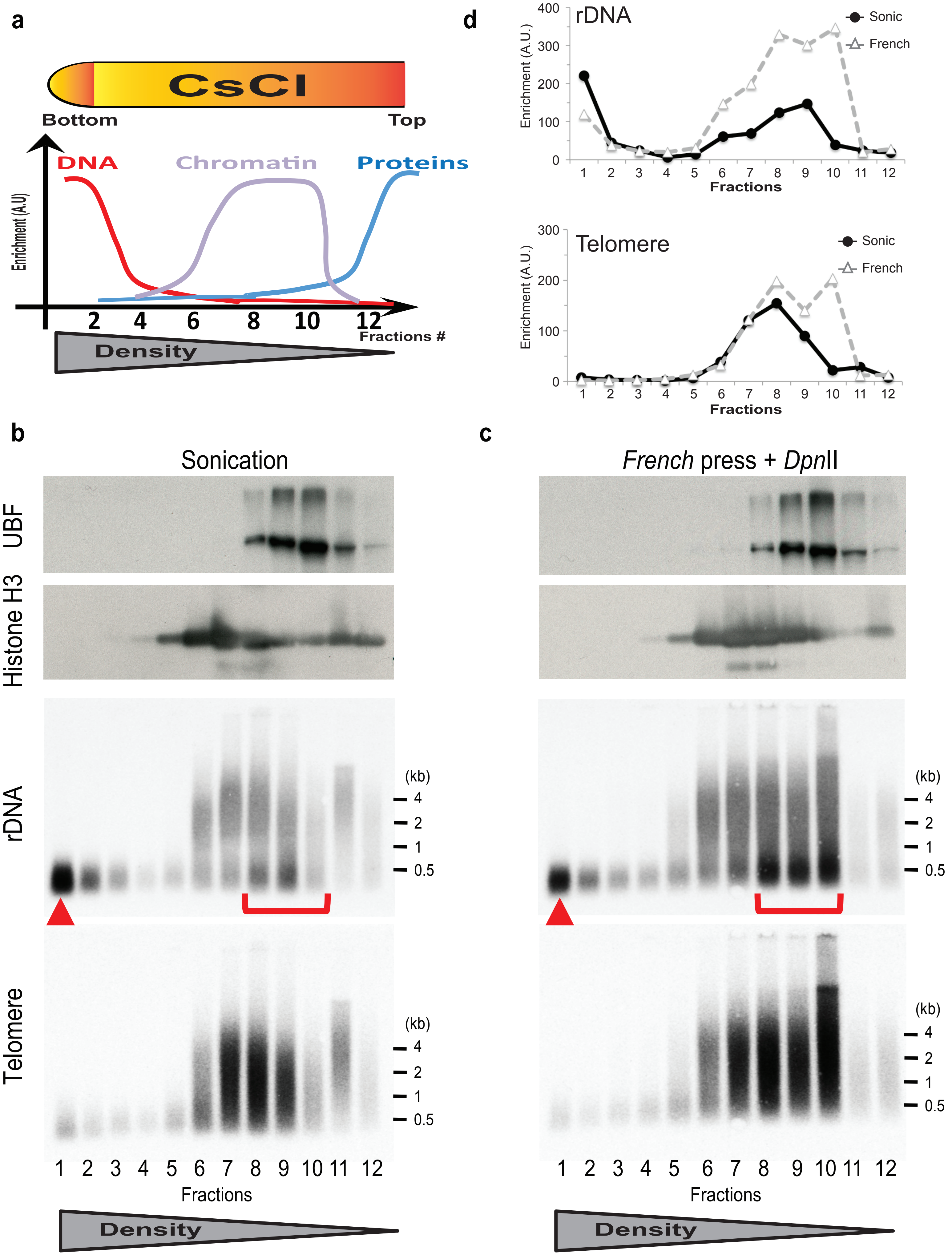
a
CsCl
Bottom
Top
DNA
Chromatin
Proteins
Enrichment (A.U)
2 4 6 8 10 12
Fractions #
Density
d
rDNA
Sonic
French
Enrichment (A.U.)
Fractions
Telomere
b
Sonication
c
French press + DpnII
UBF
Histone H3
rDNA
Telomere
(kb)
4
2
1
0.5
1 2 3 4 5 6 7 8 9 10 11 12
Fractions
Density

Figure S3

Supplementary Figure 3 | Features of crosslinked chromatin solubilized by sonication and French press.

(a) Separation principle of CsCl gradients. **(b)** Fixed chromatin solubilized by sonication and **(c)** Fixed chromatin solubilized by *French* press after DpnII digestion were fractionated by CsCl density gradient ultracentrifugation. See the Materials and Methods section for details. Proteins in each fraction were analyzed by western blotting for the rDNA transcription factor, UBF, and histone H3. The distribution of DNA fragments from rDNA promoter (rDNA) or telomere (TEL) were detected by Southern hybridization with radiolabeled 5' ETS probe or an oligonucleotide probe composed of $(TTAGGG)_9$ Red brackets in fraction 8-10 of (b) and (c) show which species are likely to be pulled down as chromatin by ePICh probes. The red arrowhead in Fraction 1 points to a density where naked DNA equilibrates without any chromatin protein. **(d)** Distribution of rDNA and telomere DNA fragments in each gradient fraction with chromatin solubilized by sonication (circle and solid line) and *French* press (triangle and dot line). Arbitrary unit (A. U.) reflecting relative amount of DNA in each fraction are indicated.

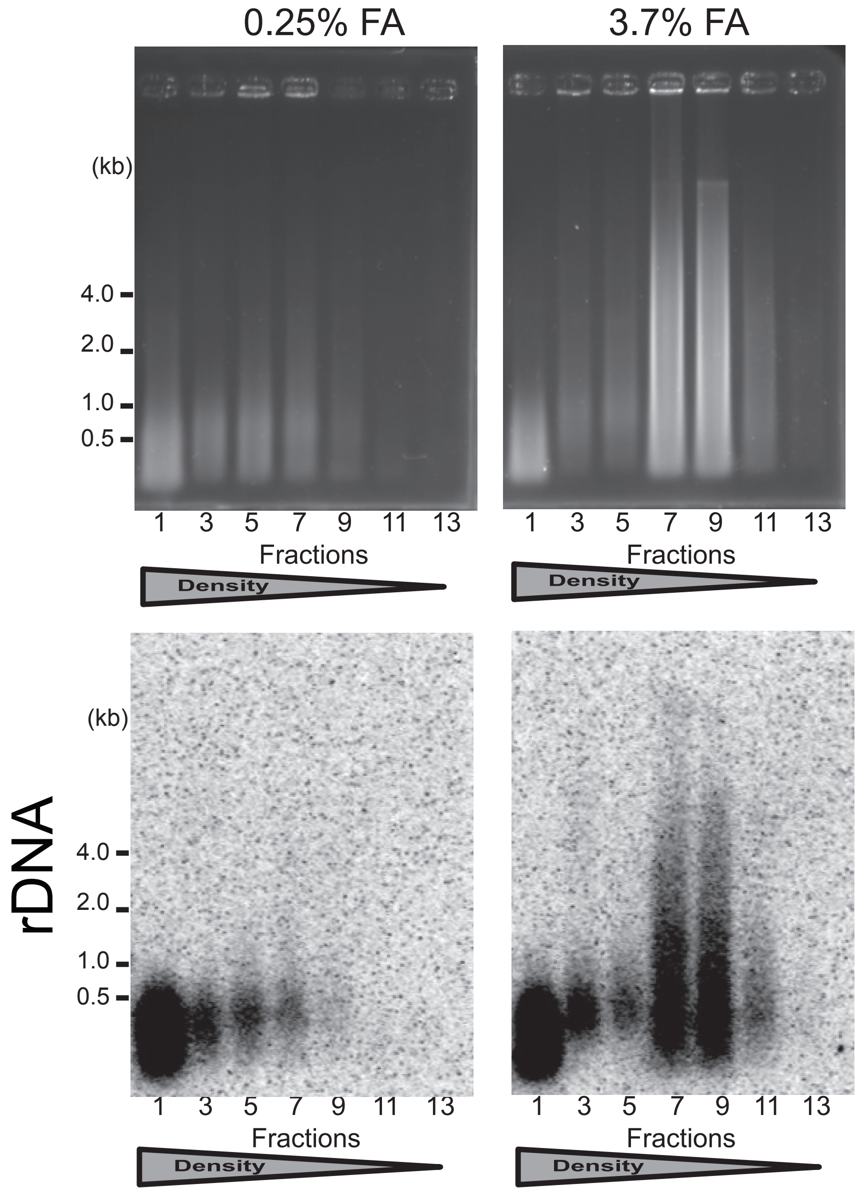
0.25% FA
3.7% FA
(kb)
4.0
2.0
1.0
0.5
1 3 5 7 9 11 13
Fractions
Density
1 3 5 7 9 11 13
Fractions
Density
rDNA
(kb)
4.0
2.0
1.0
0.5
1 3 5 7 9 11 13
Fractions
Density
1 3 5 7 9 11 13
Fractions
Density

Figure S4

Supplementary Figure 4 | Features of chromatin crosslinked with low amounts of formaldehyde 3.7% & 0.25% formaldehyde-crosslinked chromatin were fractionated by CsCl density gradient ultracentrifugation. The presence of rDNA in each fraction was monitored by Southern blotting.

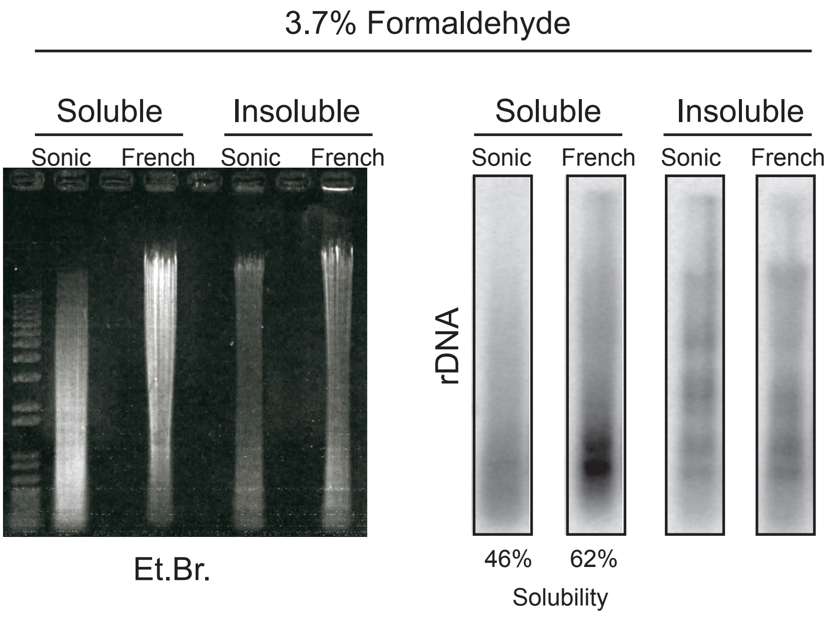
3.7% Formaldehyde
Soluble
Insoluble
Sonic
French
Sonic
French
Et.Br.
Soluble
Insoluble
Sonic
French
Sonic
French
rDNA
46%
62%
Solubility

Figure S5

Supplementary Figure 5 | Solubility of crosslinked-chromatin . rDNA from soluble (supernatant) and insoluble (pellet) fractions after sonication or *French* press was analyzed by agarose gel electrophoresis after crosslinking reversal. DNA was detected by EtBr staining (left panel) and rDNA was quantified by Southern blotting with probes specific for the 5’ETS region.

a

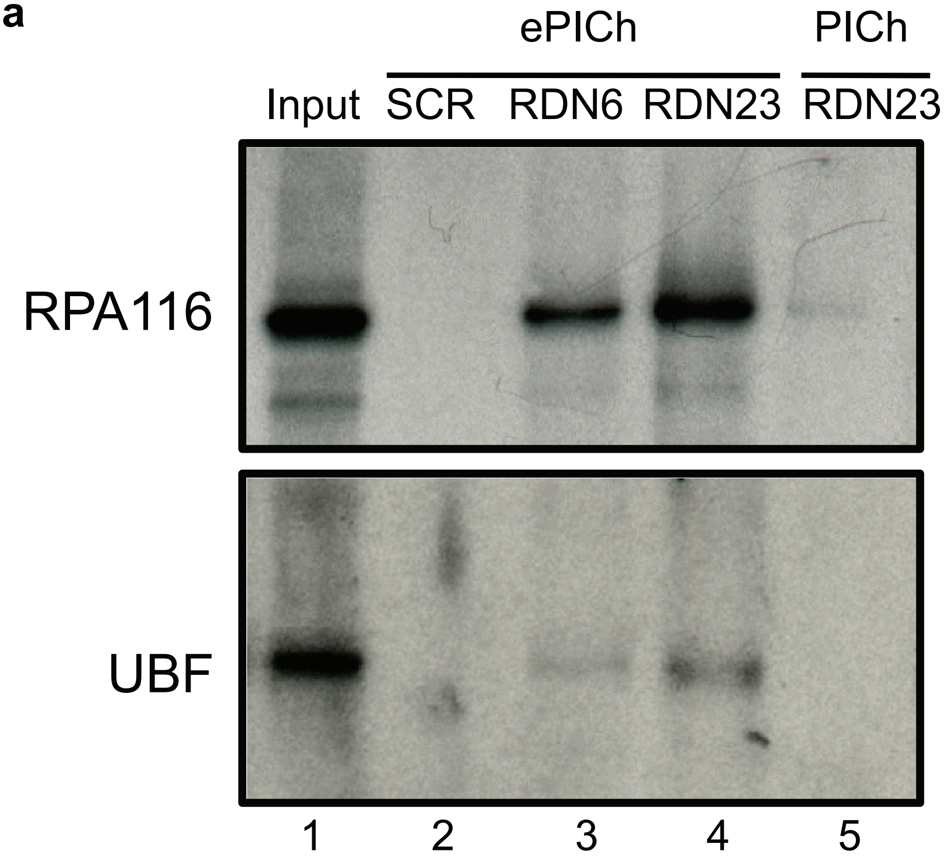

b

	ePICh			PICh
	SCR	RDN6	RDN23	RDN23
5'ETS	0	1.8	4.1	1.4
T0	0	0.4	3.1	0.3

(%Input)

Figure S6

Supplementary Figure 6 | Western blot analysis of RPA116 and UBF and quantification of ribosomal DNA yield in materials from ePICh and PICh. (a) ePICh with RDN6 probes (lane 3), with RDN23 probes (lane 4), and standard PICh (sonication and no *DpnII* restriction digestion) with RDN23 probes (lane 5) were performed in parallel. As a negative control, ePICh with SCR probe was also performed (lane 2). **(b)** Quantification of ribosomal DNA containing 5'ETS region, the TTF-1 binding site in eluates relative to Input (%Input) by quantitative PCR with primer chop 1 & 2 (TTF-1) or chop 3 & 4 (5'ETS).

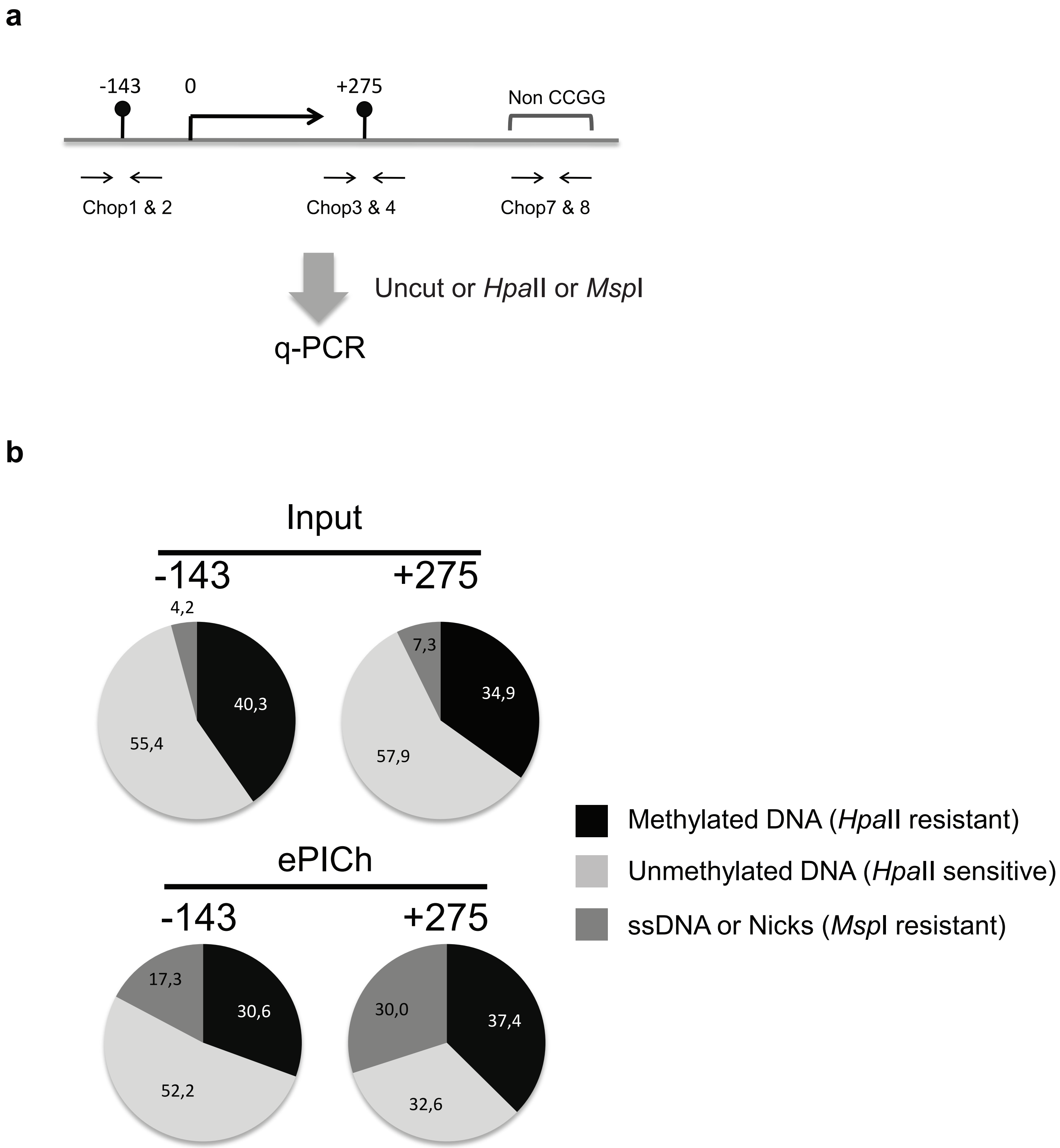

Figure S7 **Figure S6**

Supplementary Figure 7 | Measurement of the population of methylated rDNA and hypomethylated rDNA in ePICh material. (a) Primer positions around the rDNA promoter. Primer chop1 & 2 flank CCGG sequence 143 bp upstream of the TSS and primer chop 3&4 flank CCGG site 275 downstream of the TSS. **(b)** Percentage of methylated rDNA relative to hypomethylated rDNA. HpaII resistant rDNA (Methylated DNA), HpaII sensitive rDNA (Unmethylated DNA) and MspI resistant rDNA (ssDNA or DNA with nicks) was relatively quantified by q-PCR with chop 1 & 2 or chop 3 & 4. Chop5 & 6 PCR was used for the normalization of total rDNA amounts. DNA used is from ePICh with the RDN6 probe set.

	Total number of peptides		
**PROTEIN**	**RDN6**	**RDN23**	**SCR**
POLR1A (A190)	37	72	0
POLR1B (A135)	29	49	0
POLR1C (AC40)	11	14	2
POLR1D (AC19)	0	0	0
ZNRD1 (A12.2)	0	0	0
POLR1E (A49)	13	21	0
CD3EAP (A34.5)	17	32	0
TWISTNB(A43)	5	6	0

Table S1

Supplementary Table 1 | Protein list of RNA polymerase specific subunits.

	Total number of peptides			
**PROTEIN**	**RDN6**	**RDN23**	**SCR**	**Alternative name**
TAFI48	6	13	0	a subunit of SL1
TAFI63	10	15	0	a subunit of SL1
TAFI110	16	20	0	a subunit of SL1
TAFID	1	2	0	a subunit of SL1
TBP	0	0	0	TATA binding
TIF-IA	0	0	0	Rrn3
UBTF	163	174	15	UBF
TTF-1	13	23	0	Ttf1

Table S2

Supplementary Table 2 | Protein list of Pre-initiation complex for RNA pol I transcription.
